# Anti-Infective Secondary Metabolites of the Marine Cyanobacterium *Lyngbya* Morphotype between 1979 and 2022

**DOI:** 10.3390/md20120768

**Published:** 2022-12-07

**Authors:** Diaa T. A. Youssef, Shatha J. Mufti, Abeer A. Badiab, Lamiaa A. Shaala

**Affiliations:** 1 Department of Natural Products, Faculty of Pharmacy, King Abdulaziz University, Jeddah 21589, Saudi Arabia; 2 Natural Products Unit, King Fahd Medical Research Center, King Abdulaziz University, Jeddah 21589, Saudi Arabia; 3 Suez Canal University Hospital, Suez Canal University, Ismailia 41522, Egypt

**Keywords:** marine cyanobacteria, *Lyngbya* morphotype, secondary metabolites, antibacterial, antifungal, antiparasitic, antiviral, molluscicidal, anti-diatom, mode of action

## Abstract

Cyanobacteria ascribed to the genus *Lyngbya* (Family Oscillatoriaceae) represent a potential therapeutic gold mine of chemically and biologically diverse natural products that exhibit a wide array of biological properties. Phylogenetic analyses have established the *Lyngbya* ‘morpho-type’ as a highly polyphyletic group and have resulted in taxonomic revision and description of an additional six new cyanobacterial genera in the same family to date. Among the most prolific marine cyanobacterial producers of biologically active compounds are the species *Moorena producens* (previously *L. majuscula,* then *Moorea producens*)*, M. bouillonii* (previously *L. bouillonii*), and *L. confervoides*. Over the years, compounding evidence from in vitro and in vivo studies in support of the significant pharmaceutical potential of ‘*Lyngbya*’-derived natural products has made the *Lyngbya* morphotype a significant target for biomedical research and novel drug leads development. This comprehensive review covers compounds with reported anti-infective activities through 2022 from the *Lyngbya* morphotype, including new genera arising from recent phylogenetic re-classification. So far, 72 anti-infective secondary metabolites have been isolated from various *Dapis*, *Lyngbya*, *Moorea*, and *Okeania* species. These compounds showed significant antibacterial, antiparasitic, antifungal, antiviral and molluscicidal effects. Herein, a comprehensive literature review covering the natural source, chemical structure, and biological/pharmacological properties will be presented.

## 1. Introduction

Infectious diseases, also known as transmissible diseases or communicable diseases, are illnesses caused by a harmful pathogen. Infections can be a result of a wide range of pathogens, such as bacteria, viruses, parasites, and fungi. The immune system of the host is always responsible for the fight against the cause of infection. Anti-infective drugs have improved the way for modern medicine and saved the life of millions of people. They are considered a vital group of drugs in this regard and have contributed significantly to the improvement of lifestyle and the rise in life expectation over the past decades [1,2]. Natural products contributed significantly to the major group of current anti-infective drugs [3]. However, for a long time, the pharmaceutical industry has ignored the search for natural product-derived and novel anti-infective drug discovery [4]. This fact resulted in a situation where illness with antibiotic resistant microbes regularly cannot be effectively treated [5]. Illnesses with Gram-negative bacteria and a developed treatment resistance, such as *Enterobacter* and *Pseudomonas*, are considered serious issues, while a few new antibiotics are under development [6].

The development of Antimicrobial Resistance (AMR) and multi-drug resistance to current therapeutics represent a serious issue for the patients, health care system and for the economy worldwide [7,8,9,10,11,12,13,14,15,16,17]. In addition, viral infections are spreading worldwide rapidly [18,19,20,21,22,23,24,25,26] and are considered a threat to the healthy community and represent additional economic burden to the concerned countries worldwide [18,19,20,21,22,23,24,25,26]. Therefore, it is of an urgent need to develop new drugs for the combat against AMR and viral infections.

Recently, the pandemic of coronavirus (COVID-19) resulted in hundreds of thousands of deaths in several countries around the world. SARS-CoV-2, a newly found coronavirus strain, causes COVID-19, an infectious respiratory illness. Remdesivir is being used in conjunction with the anti-inflammatory drugs dexamethasone or baricitinib as treatment for this coronavirus. Because of Remdesivir’s side effects, which include respiratory failure, hypokalemia, and headaches, new medications for effective COVID-19 therapy are urgently needed [27]. Therefore, more research is needed to develop powerful antivirals as alternative treatments that can improve the management of epidemics and eventually lead to their elimination.

Water occupies more than 70% of our Blue Planet. Distinct environments from oceans and seas to freshwater lakes, wetlands, ponds, rivers and streams, all of which exhibit diverse ecosystems, allowing the inhabitation of species with distinct characteristics, differing from terrestrial organisms. The advent of SCUBA and submersible technologies has enabled scientists to explore the plethora of marine natural products created by biologically diverse marine organisms and to investigate their biological activities. The lack of cures and treatments for many diseases compels the scientific community, academia and industry to search untapped avenues and focus on underexplored territories, which makes marine drug discovery a prime target for pharmaceutical research. Marine drug discovery is still considered in its infancy, and the full therapeutic potential of marine natural products is yet to be realized.

In this regard, scientists have investigated the biological activities of numerous natural products isolated from marine organisms in order to search for any promising pharmacological properties that may be utilized in the development of viable treatment modalities. Some of the marine organisms most widely studied for their natural products are benthic filamentous cyanobacteria collected pantropically.

For more than three billion years, cyanobacteria inhabited the earth, representing one of the eldest known organisms [28]. Cyanobacteria are diverse in terms of their physiology, metabolism and morphology. They inhabit all environments worldwide, including freshwater, marine environment and extreme habitats [29]. The production of highly potent/toxic secondary metabolites is considered as an evolutionary strategy for cyanobacteria to survive from planktivores or other ecological competitors [30,31]. It was noticed that a significant portion of the cyanobacterial secondary metabolites in the literature possessed cytotoxic activity [32]. Certainly, cytotoxicity is still today a noticeable activity of cyanobacteria-derived compounds.

Specimens characterized by relatively large filaments of discoid cells within a distinct sheath and lacking nitrogen-fixing heterocyst cells have routinely been assigned to the genus *Lyngbya* (Family Oscillatoriaceae, Order Oscillatoriales). This traditional morphology-based taxonomic identification has underestimated the biological diversity of filamentous marine cyanobacteria [33] leading to more than 500 marine-derived compounds being ascribed to the single genus *Lyngbya*. While this *Lyngbya* ‘morpho-type’ has evolved to additionally include the new genera *Dapis* [34], *Limnoraphis* [35]*, Moorena* (previously *Moorea*) [36]*, Microseira* [37] and *Okeania* [38], and further taxonomic revisions are in progress, it remains a group with tremendous therapeutic potential.

Currently, there are 17 marketed marine-derived compounds or analogs, or derivatives therefrom and an additional 34 drug candidates in different phases of clinical trials (I, II, and III) [39]. Astonishingly, amongst the 17 marketed compounds, there are 5 compounds (29%) of cyanobacterial/molluscs origin. Further, among the 34 drug candidates in different clinical phases (I, II, and II), there are 23 compounds (67%) of cyanobacterial/molluscs origin [39]. This fact makes cyanobacteria/molluscs the main source of marine derived marketed drugs and drug leads under development today. Since all marketed drugs and drug candidates in clinical phases are targeting different types of cancer, more research focus from the academia and industry should be directed to combat microbial resistance and viral infections worldwide.

A literature search was conducted using the keywords “*Lyngbya*”, “*Moorea*”, “*Moorena*”, “*Neolyngbya*”, “*Okeania*”, “*Limnoraphis*”, and “*Dapis*”, on SciFinder, resulting in 2956 hits, including 2423 for *Lyngbya,* 480 for *Moorea*, 35 for *Okeania*, 9 for *Moorena*, 3 for *Neolyngbya*, 3 for *Limnoraphis*, and 3 for *Dapis*, some of which were duplicated between the databases MEDLINE and CAplus. Results showed that, while mostly cytotoxic secondary metabolites have been described from *Lyngbya* morphotype, there are also a substantial number of these compounds with anti-infective properties.

This comprehensive review addresses all antibacterial, antifungal, antiviral, antiparasitic, molluscicidal and anti-diatom activities isolated from the marine cyanobacterium *Lyngbya* morpho-type. The review will focus on the source organisms, geographical locations of the studied species, chemical diversity of the isolated compounds and associated biological activities and their mode of action, if any. About 72 compounds belonging to a diverse group of chemical classes were reported from *Lyngbya* morpho-type over 43 years between 1979 and 2022.

## 2. Collected Species and Geographical Locations

The first report about an anti-infective compound from the genus *Lyngbya* was published in 1979 [40]. Between then and 2022, a total of about 72 secondary metabolites with anti-infective properties were reported from 11 morphotype species belonging to the genus *Lyngbya* (Table 1).

**Figure 1:**
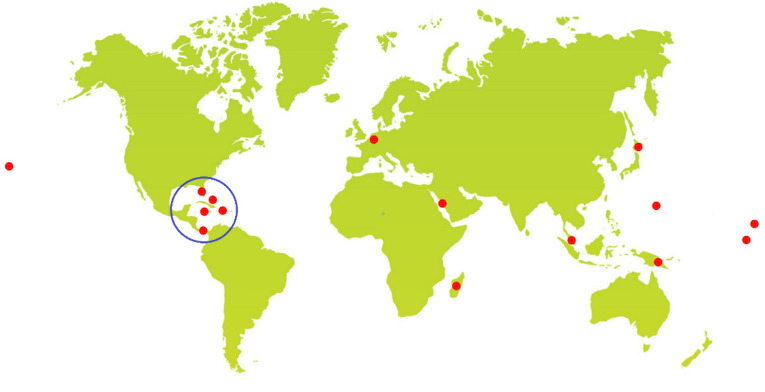
A map with red dots indicating collection locations for *Lyngbya*-morphotype described in this review.

Interestingly, more than 50% of the reported compounds come from *L. majuscula* (Table 1). These species have been collected from locations around the world, focused on tropical regions (Figure 1).

**Figure 2:**
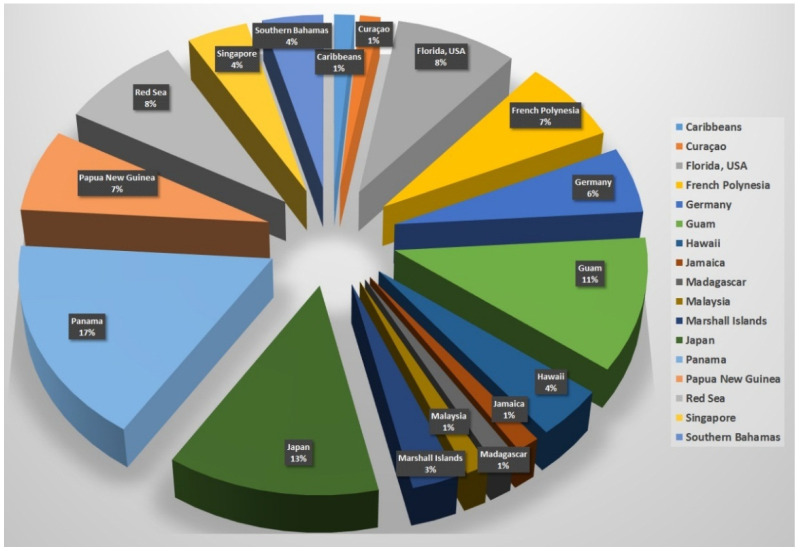
Number of reported anti-infective compounds related to collection site.

A notable number of reported compounds (17%) comes from species that collected in Panama, making this place a diverse and rich location for collecting cyanobacterial strains. Japan comes in the second place with 9 compounds (13%) followed by Guam with 8 compounds (11%). In third place, Florida (USA), Red Sea and Japan, each comes with 6 compounds (8%). (Figure 2).

These data indicate that genus *Lyngbya* continues to be a rich source of secondary metabolites that are new to science and suggest potential locations for further discovery.

## 3. Compounds with Antibacterial Activities

**Figure 3:**
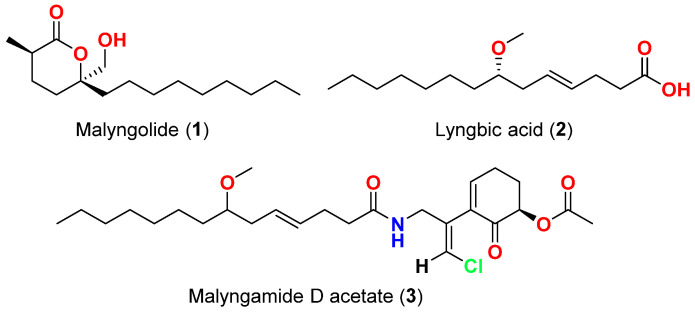
Chemical structures of compounds **1**–**3**.

Among the diverse bioactivities that *Lyngbya* secondary metabolites have displayed is the activity against bacteria. In 1979, malyngolide (**1**) (Figure 3), a δ-lactone was reported from Hawaiian *Lyngbya majuscula* in Kahala Beach, showed effective antibacterial activity against *Mycobacterium smegmatis* and *Streptococcus pyogenes* and was less active against *Staphylococcus aureus* and *Bacillus subtilis*, and inactive towards *Enterobacter aerogenes*, *Escherichia coli*, *Pseudomonas aeruginosa*, *Salmonella enteritidis*, and *Staphylococcus marcescens* [40].

In 1987, the fatty acid (-)-7(*S*)-methoxytetradec-4(*E*)-enoate (lyngbic acid) (**2**) (Figure 3), was purified from *Moorea producens* collected at the Red Sea, near Jeddah, Saudi Arabia [41], displayed antibacterial activity against *S. aureus* and *B. subtilis* [41].

The related amide of lyngbic acid, malyngamide D acetate (**3**) (Figure 3), which were isolated from Caribbean *L. majuscula* in Isla Guayacan, Puerto Rico in 1987, displayed slight activity against *S. aureus* [42].

**Figure 4:**
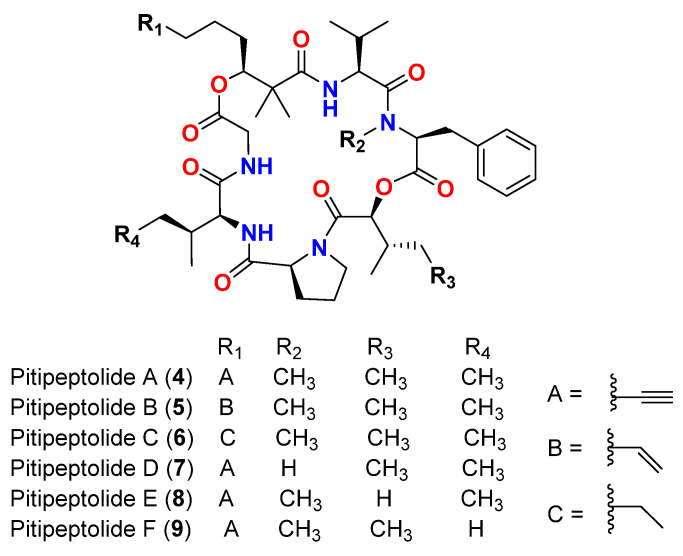
Chemical structures of compounds **4**–**9**.

In 2001, the cyclic depsipeptides pitipeptolides A (**4**) and B (**5**) (Figure 4) are reported from *L. majuscula* collected in Piti Bomb Holes, Guam [43]. The compounds displayed moderate activity against *Mycobacterium tuberculosis* strains (ATCC 25177 and ATCC 35818) in the antimycobacterial diffusion susceptibility assay. Pitipeptolide A (**4**) gave a diameter of growth inhibition zone for ATCC 25177 strain equivalent to 25 and 10 mm, and for ATCC 35818 strain equivalent to 15 and 9 mm upon treatment with 100 and 25 µg, respectively. Pitipeptolide B (**5**) gave a diameter of growth inhibition zone for ATCC 25177 strain equivalent to 30 and 15 mm, and for ATCC 35818 strain equivalent to 15 and 10 mm upon treatment with 100 and 25 µg, respectively. For comparison, treatments with 25, 5 and 1 µg of streptomycin resulted in superior activity, giving diameters of 50, 15 and 0 mm, respectively, for ATCC 25177 strain, and 55, 33 and 10 mm, respectively, for ATCC 35818 [43].

Ten years later, in 2011, pitipeptolides C-F (**6**–**9**) (Figure 4) are reported from *L. majuscula* in Piti Bomb Holes, Guam. The study showed that pitipeptolide F (**9**) was the most potent compound in the antimycobacterial disc diffusion assay (*M*. *tuberculosis* ATCC 25177 strain) [44]. Treatment with pitipeptolides A-F (**4**–**9**) resulted in inhibition zones of 28, 30, 26, 10, 21 and 40 mm, respectively, at 100 µg, 23, 24, 21, 0, 15 and 30 mm, respectively, at 50 µg, and 9, 14, 18, 0, 0 and 10 mm, respectively, at 10 µg. For comparison, streptomycin gave 40, 30 and 0 mm inhibition zone upon using 10, 5 and 1 µg treatment, respectively [44].

SAR studies revealed that the *N*-methylation in the Phe unit is essential for both cytotoxic and antibacterial activities, whereas the π system in the fatty acid unit was found to be one of the important structural features for the cytotoxic activity in mammalian cells, but it was not required for antibacterial activity. Furthermore, decreasing the hydrophobicity of certain units (2-Hydroxy 3-methyl pentanoic acid (Hmpa) → 2-Hydroxy isovaleric acid (Hiva) and Ile → Val) caused a reduction in the anticancer activity (as seen with pitipeptolides E and F), while on the other hand resulted in an increase in antimycobacterial potency (particularly pitipeptolide F) [44].

**Figure 5:**
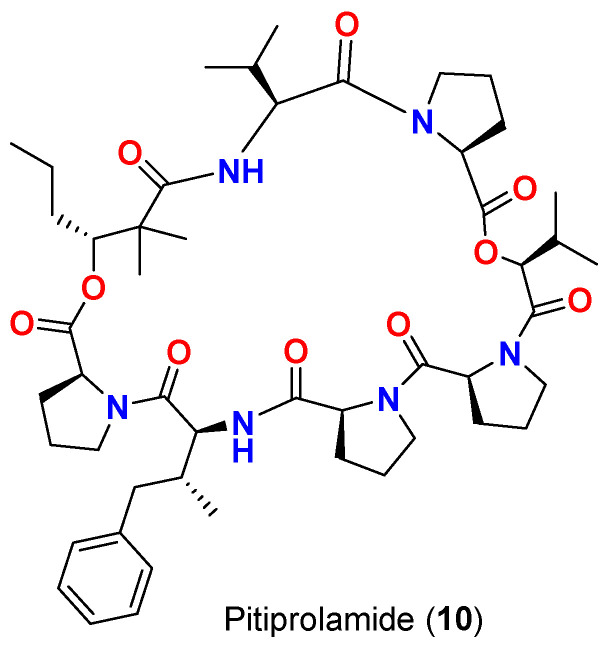
Chemical structure of compound **10**.

Pitiprolamide (**10**) (Figure 5), a dolastatin 16 analog and a proline rich cyclic depsipeptide was purified in 20111 from the same Guamanian cyanobacterium *Lyngbya majuscula* collected at Piti Bomb Holes, displayed weak antimycobacterial effect against *M. tuberculosis* (ATCC 25177 strain) starting at 50 μg in a disk diffusion assay. The compound displayed zone of inhibition of 23, 13 and 0 mm after 100, 50 and 10 µg treatment. Also, the compound exerted weak antibacterial activity against *Bacillus cereus* (ATCC 10987 strain) starting at 1 μM in a microtiter plate-based assay with an approximate IC_50_ value of 70 μM and lacked the activity against *S. aureus* and *P. aeruginosa* [45].

**Figure 6:**
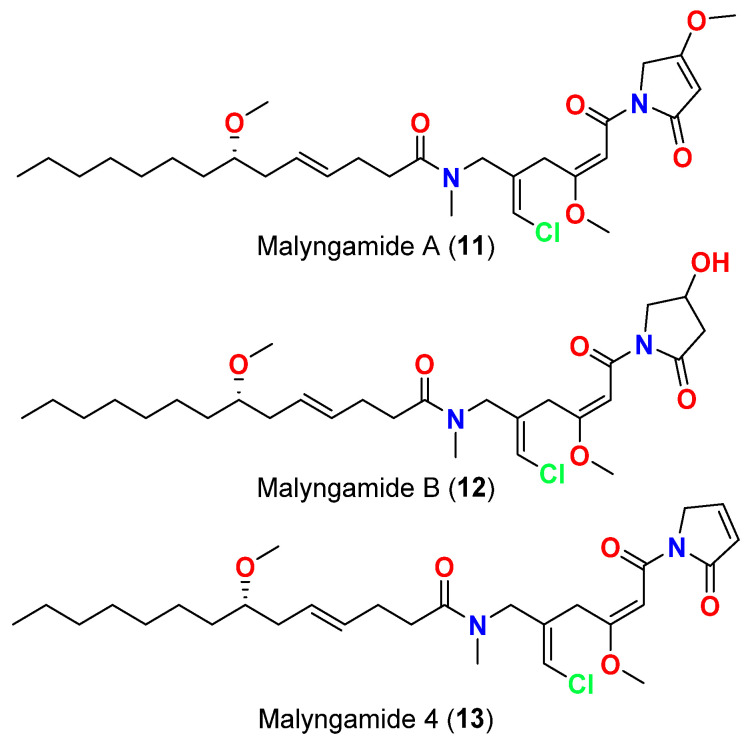
Chemical structures of compounds **11**–**13**.

Table 2 that purified in 2013 from the cyanobacterium *M. producens* collected at the Red Sea, near Jeddah, Saudi Arabia, significantly inhibited the growth of *M. tuberculosis* H_37_Rv in vitro (65% inhibition) at a concentration of 12.5 μg/mL, while the chlorinated lipopetides malyngamides A (**11**), B (**12**) and 4 (**13**) (Figure 6) (obtained from the same cyanobacterial collection) displayed much weaker antimycobacterial activity at the same tested concentration, which was deemed as ineffective (18, 10 and 17% inhibition, respectively) [41]. This result suggests the importance of a terminal free carboxylic acid moiety for the antimycobacterial effect.

**Figure 7:**
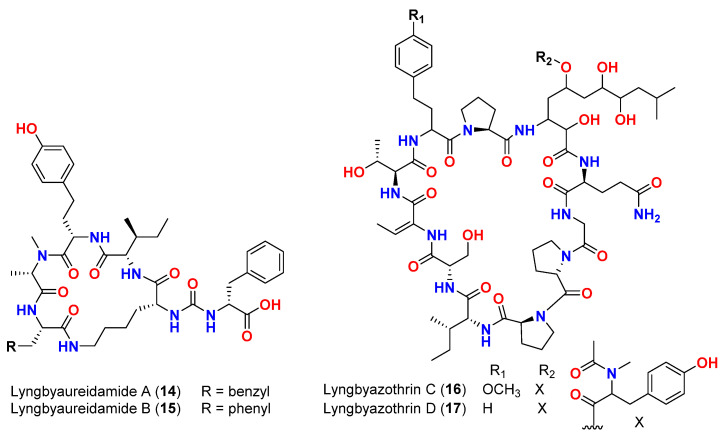
Chemical structures of compounds **14**–**17**.

Another group of antimicrobial natural products is the cyclic undecapeptides lyngbyazothrins A and B (**14** and **15**) and C and D (**16** and **17**) (Figure 7), which were isolated as binary mixtures from *Lyngbya* sp. 36.91 SAG (Culture Collection of Algae, Gottingen, Germany) in 2009. The mixture of lyngbyazothrins A and B (**14** and **15**) showed minimal antibacterial activity against *Micrococcus flavus* SBUG 16 in the agar diffusion disk (100 μg/disk: 8 mm diameter of inhibition zone). The mixture of lyngbyazothrins C (**16**) and D (**17**) showed modest activity against *B. subtilis* SBUG 14 (25 μg/disk: 18 mm), *E. coli* ATCC 11229 (100 μg/disk: 18 mm), and *E. coli* SBUG 13 (100 μg/disk: 15 mm) and low activity against *P. aeruginosa* ATCC 27853 (100 μg/disk: 8 mm) and *Serratia marcescens* SBUG 9 (200 μg/disk: 8 mm). When used at the same concentrations, the lyngbyazothrins A and B (**14** and **15**) mixture lacked activity against the aforementioned strains, which suggests that the linkage of the acyl residue at C-5 of the 3-amino-2,5,7,8-tetrahydroxy-10-methylundecanoic acid (Aound) unit may be responsible for the antimicrobial activity [46].

**Figure 8:**
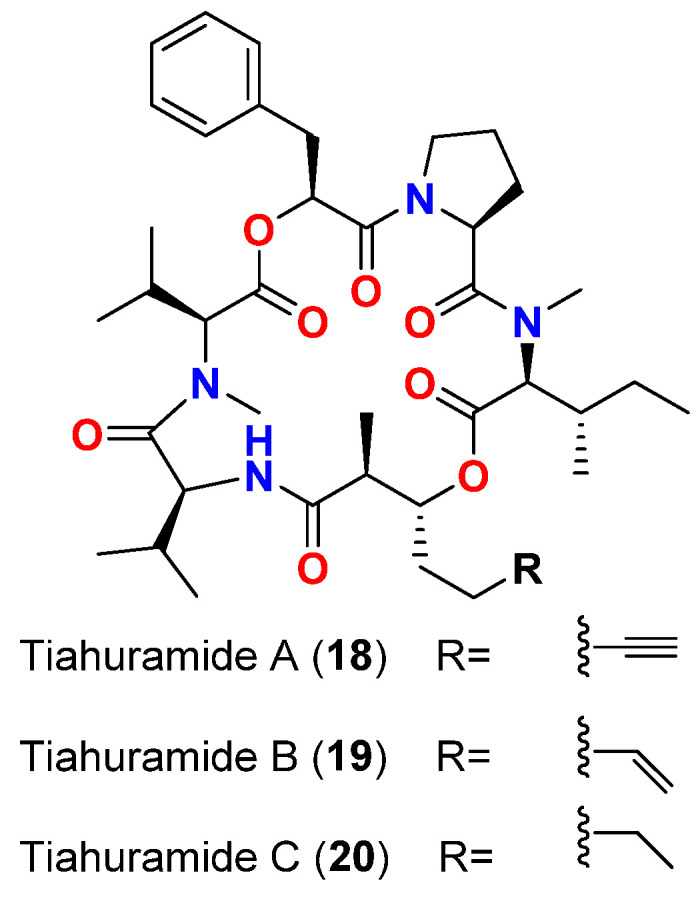
Chemical structures of compounds **18**–**20**.

The intriguing cyclic depsipeptides, tiahuramides A-C (**18**–**20**) (Figure 8), are isolated in 2018 from *L. majuscula* collected at Tiahura sector, Moorea Island in French Polynesia, displayed growth inhibitory activities on opportunistic marine pathogenic bacteria (*Aeromonas salmonicida* (CIP 103209T strain), *Vibrio anguillarum* (CIP 63.36T), and *Shewanella baltica* (CIP 105850T)) and terrestrial bacteria (*E. coli* (CIP 54.8) and *Micrococcus luteus* (CIP A270)). The MIC values against *A. salmonicida*, *V. anguillarum*, *S. baltica*, *E. coli* and *M. luteus* were as follows: 27, 33, >50, 35 and 47 μM, respectively, for tiahuramide A; 9.4, 8.5, 22, 12 and 29 μM, respectively, for tiahuramide B; and 6.7, 7.4, 16, 14 and 17 μM, respectively, for tiahuramide C. As evidenced by the MIC values, tiahuramide C (**20**) exhibited the greatest antibacterial potency followed by tiahuramide B (**19**), whereas tiahuramide A (**18**) was the least active among this series of compounds [47].

Table 2 summarizes all compounds with reported antibacterial effects, their sources and collection sites as well as the targeted bacteria and observed effects.

## 4. Compounds with Anti-Swarming and Anti-Quorum Sensing Activities 

Some compounds exert their antibacterial activities by inhibiting swarming, a mechanism used by bacteria to spread across surfaces supplied with nutrients through the use of rotating flagella in order to speed their growth [48,49].

**Figure 9:**
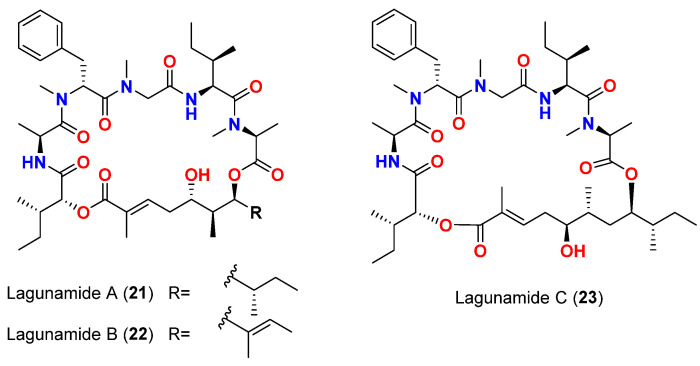
Chemical structures of compounds **21**–**23**.

Lagunamides A-C (**21**–**23**) (Figure 9), cyclic depsipeptides purified in 2010 and 2011 from *L. majuscula* found in Pulau Hantu Besar, Singapore, exhibited moderate to weak anti-swarming activities against the Gram-negative bacterial strain *P. aeruginosa* PA01 (62, 56 and 49% compared to control, respectively) when tested at 100 ppm; *P. aeruginosa* PA01 (62, 56 and 49% compared to control, respectively) when tested at 100 ppm [50,51].

On the other hand, other compounds exert their antimicrobial activities by interfering with quorum sensing (QS), a mechanism that is responsible for the regulating of the bacterial gene expression in response to fluctuations in cell-population density [52,53].

**Figure 10:**
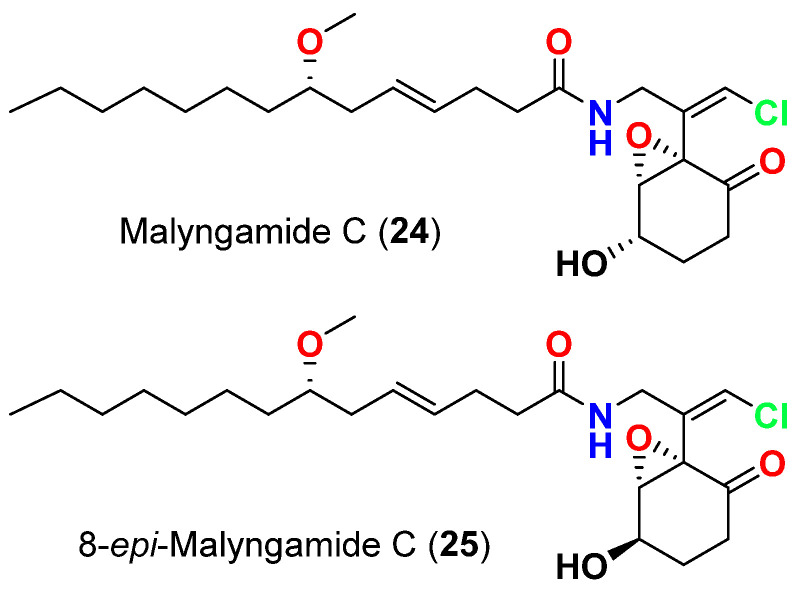
Chemical structures of compounds **24** and **25**.

In 2010, malyngamide C (**24**) and 8-*epi*-malyngamide C (**25**) (Figure 10) are reported from *L. majuscula* collected in Bush Key, Dry Tortugas, Florida, displayed activity against the QS reported pSB1075, which expresses LasR and responds to 3-oxo-C_12_-HSL (*N*-3-oxo-dodecanoyl-L-homoserine lactone). Using concentrations of both compounds that did not actually inhibit bacterial cell growth (10, 100 and 1000 µM) resulted in reducing 3-oxo-C_12_-HSL signalling in the QS reporter [54].

Malyngolide (**1**), an antibiotic isolated from *L. majuscula* in South Florida, inhibited violacein pigment production by *Chromobacterium violaceum* CV017 in the QS bioassay. Effective concentrations ranged from 0.07 to 0.22 mM, with an EC_50_ value of 0.11 mM, and the growth of the *C. violaceum* reporter strain was not inhibited even at the higher concentration used (0.22 mM). In the presence of 14 µM of 3-oxo-C_12_-HSL, malyngolide (**1**) inhibited responses of the *lasR*^+^P_lasI_-*luxCDABE* reporter pSB1075 when used at concentrations ranging from 3.57 to 57 µM (EC_50_ = 12.2 µM) without affecting bacterial growth. At these concentrations, malyngolide (**1**) also significantly reduced the production of elastase by *P. aeruginosa* PAO1, which is an extracellular enzyme regulated by 3-oxo-C_12_-HSL and LasR, with an EC_50_ value of 10.6 µM. At higher concentrations of malyngolide, elastase production was inhibited to the level observed in the QS mutant of *P. aeruginosa* JP2. It is worth mentioning that a decline in the activity of malyngolide was noticed upon storing it in plastic instead of glass vials [55].

**Figure 11:**
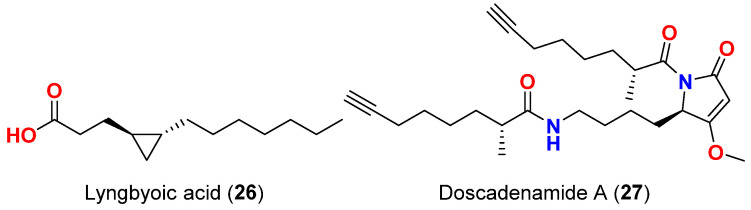
Chemical structures of compounds **26** and **27**.

Another disruptor of QS in *P. aeruginosa* is lyngbyoic acid (**26**) (Figure 11), a small cyclopropane-containing fatty acid isolated was reported in 2019 from *L. majuscula* collected at various sites in Florida. The compound was evaluated against four reporters based on different acylhomoserine lactone (AHL) receptors (LuxR, AhyR, TraR and LasR), and LasR turned out to be the most reported being affected by lyngbyoic acid (**26**). It also reduced the production of pyocyanin and elastase (LasB) both on the protein and transcript level in wild-type *P. aeruginosa*, and directly inhibited LasB enzymatic activity with a *K*_i_ of 5.4 mM, without affecting bacterial growth [56].

Finally, in 2019, doscadenamide A (**27**) (Figure 11), was isolated from *M. bouillonii* collected in Fingers Reef, Apra Harbor, Guam, displayed QS agonistic activities in a LasR-dependent manner. Doscadenamide A and the QS signaling molecule 3-oxo-C_12_-HSL share structural similarities as they both contain a five-membered ring core and long alkyl side chain. Doscadenamide A activated the 3-oxo-C_12_-HSL-responsive reporter plasmid pSB1075, which encodes LasR and contains a light-producing *luxCDABE* cassette expressed in *E. coli*; however, it was not able to activate the related reporter pTIM5319, which is identical to pSB1075, except for lacking the AHL-binding site LasR, thereby suggesting that doscadenamide A activates QS via the AHL-binding site. The effect of the compound was tested on wild-type *P. aeruginosa*, using effective doses of 10, 100 and 1000 µM, and maximal induction of the QS pigment pyocyanin production was observed upon usage of even the lowest concentration. Levels of pyocyanin increased after only 6 h of treatment with 10 µM of doscadenamide A, which was a comparable result with using 10 µM of the positive control 3-oxo-C_12_-HSL [57].

Table 3 summarizes all compounds with reported anti-swarming and anti-quorum sensing effects, their sources and collection sites as well as the targeted bacteria and observed effects.

## 5. Compounds with Antifungal Activities

**Figure 12:**
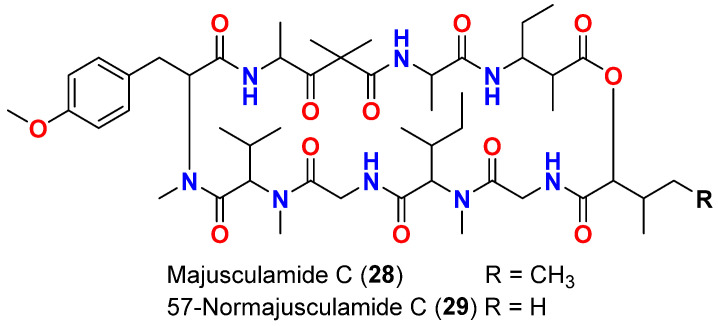
Chemical structures of compounds **28** and **29**.

Antifungal assays are among the widely used bioassays for testing the activities of natural compounds isolated from cyanobacteria. Majusculamide C (**28**) (Figure 12), a cyclic depsipeptide reported in 1984 from *L. majuscula* in Marshall Islands, inhibited the growth of a number of fungal plant pathogens such as *Phytophthora infestans* and *Plasmopora viticola,* the causative organisms of tomato late blight and grape downy mildew, respectively [58].

In 1988, 57-normajusculamide C (**29**) (Figure 12) was purified from the marine cyanobacterium *L. majuscula* collected in Marshall Islands. The compound displayed antimycotic activity against the indicator organism *Saccharomyces pastorianus* [59].

**Figure 13:**
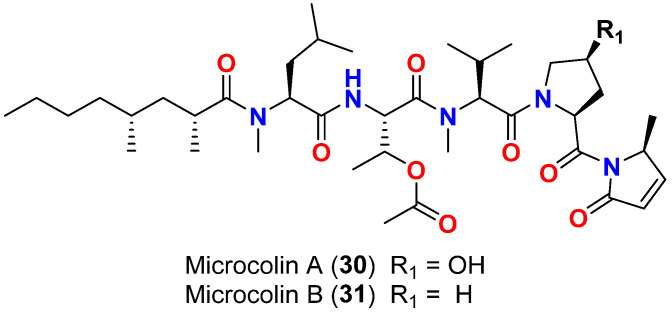
Chemical structures of compounds **30** and **31**.

Microcolins A (**30**) and B (**31**) (Figure 13), lipopeptides isolated from Floridian *L. polychroa*, showed only little activity against two strains (SIO and EBGJ) of the marine fungus *Dendryphiella salina*, which has been linked to diseases among marine algae and seagrasses, where the LD_50_ values were above 200 μg/mL in the antifungal assay. The antifungal activities of microcolins A (**30**) and B (**31**), were significantly lower than the known antifungal compound amphotericin B, which resulted in 100% inhibition of marine fungus *Dendryphiella salina* in the same assay at concentrations as low as 3.13 μg/mL [60].

**Figure 14:**
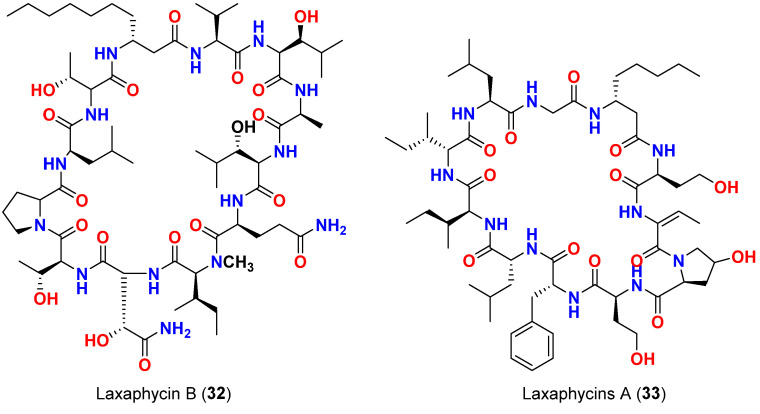
Chemical structures of compounds **32** and **33**.

The majority of natural products have been tested for their antifungal activity against *Candida albicans* as reported herein. Laxaphycin B (**32**) (Figure 14), a cyclic lipopetide reported in 1997 from *L. majuscula* in Moorea Atoll, French Polynesia, exhibited antifungal activity against *C. albicans*. Interestingly, laxaphycin A (**33**) (Figure 14), inactive by itself, exerted a synergistic effect when combined with laxaphycin B (**32**) and potentialized its antifungal activity. This unique difference in activity might be attributed to the chemical structures of the compounds. Laxaphycin A (**33**) is an undecapeptide with segregated hydrophobic and hydrophilic residues, while laxaphycin B (**32**) is a dodecapeptide with alternating hydrophobic and hydrophilic residues [61].

**Figure 15:**
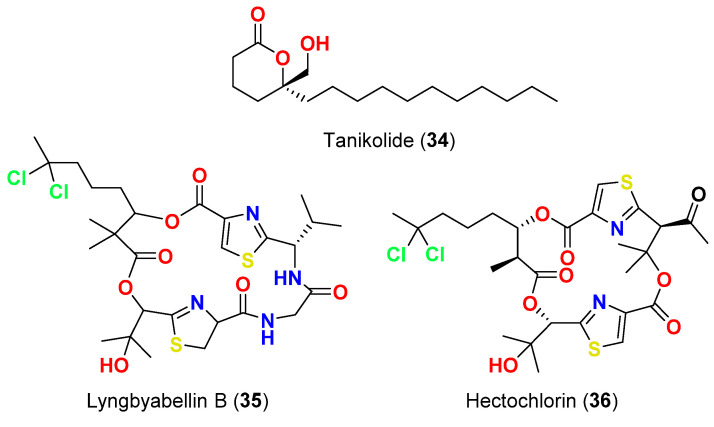
Chemical structures of compounds **34**–**36**.

Tanikolide (**34**) (Figure 15), a lipid lactone that was reported in 1999 from *L. majuscula* found in Tanikeli Island, Madagascar, showed antifungal activity towards *C. albicans* with 13 mm diameter zone of inhibition at 100 µg/disk using paper disk-agar plate methodology [62].

Lyngbyabellin B (**35**) (Figure 15), a cyclic depsipeptide that reported in 2000 from *L. majuscula* found in Dry Tortugas National Park in Florida, displayed antifungal effect towards *C. albicans* (ATCC 14053) in a disk diffusion assay with a 10.5 mm zone of inhibition at 100 µg/disk and a slight halo at 10 µg/disk [63].

In 2002, the lipopeptide hectochlorin (**36**) (Figure 15), was reported from *L. majuscula* found in both Hector Bay, Jamaica, and Boca del Drago Beach, Panama. The compound produced a 16 mm zone of inhibition at 100 µg/disk and an 11 mm zone of inhibition at 10 µg/disk against *C. albicans* (ATCC 14053) [64].

**Figure 16:**
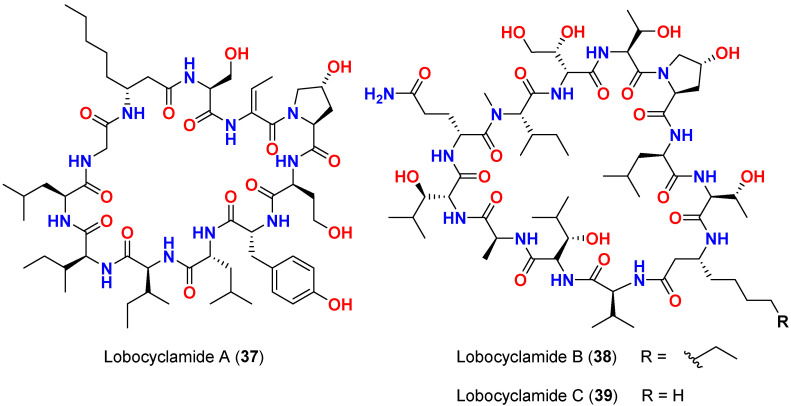
Chemical structures of compounds **37**–**39**.

In 2002, the lipopeptides lobocyclamides A–C (**37**–**39**) (Figure 16) are obtained from a cyanobacterial mat containing *L. confervoides* found in Cay Lobos, Southern Bahamas. The compounds exhibited moderate antifungal activities when tested in disk diffusion assay at 150 µg/disk against fluconazole-resistant fungus *C. albicans* 96–489 giving 7, 8 and 10 mm inhibition zones, respectively. When evaluated towards *C. glabrata*, lobocyclamide B (**38**) and C (**39**) produced 6 and 8 mm inhibition zone, respectively, at 150 µg/disk [65].

In the microbroth dilution assay against *C. albicans* 96–489, lobocyclamide A (**37**) displayed MIC value of 100 µg/mL, while lobocyclamide B (**38**) showed an MIC value of 30–100 µg/mL [65].

A mixture of lobocyclamides A (**37**) and B (**38**) exhibited significant synergism (e.g., 1:1 mixture of A and B produced a MIC of 10–30 µg/mL) with higher activity than either of the pure compounds used individually [65], a phenomenon also reported with laxaphycins A (**33**) and B (**32**) [61].

Table 4 summarizes all compounds with reported antifungal activities, their sources and collection sites as well as the targeted fungi and observed effects.

## 6. Compounds with Antiparasitic Activities

Tropical parasitic diseases can be life-threatening if not treated appropriately from an early stage. The most common tropical infectious parasite is *Plasmodium falciparum*, the causative organism of malaria. Several *Lyngbya*-derived compounds displayed inhibitory activities on this parasite.

**Figure 17:**
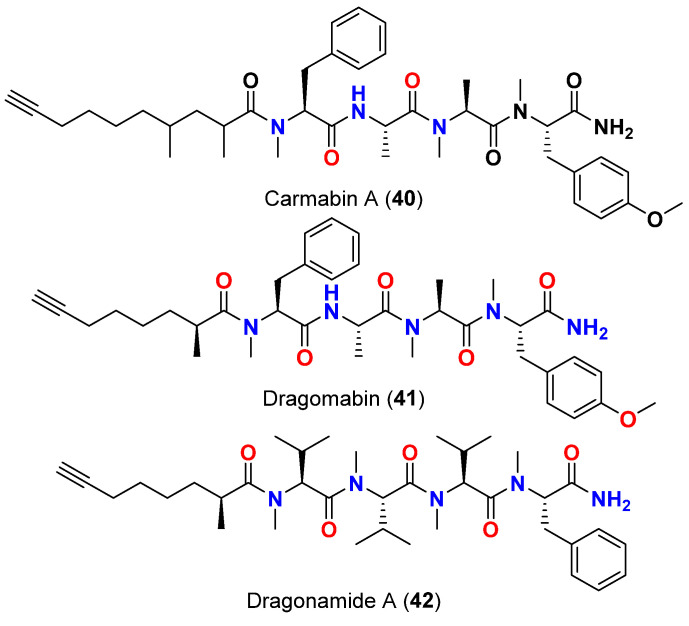
Chemical structures of compounds **40**–**42**.

Carmabin A (**40**), dragomabin (**41**) and dragonamide A (**42**) (Figure 17), are linear alkynoic lipopeptides are reported in 2007 from *L. majuscula* that was collected from Isla Bastimentos in Bocas del Toro, Panama, possessed good antimalarial activities against a chloroquine-resistant strain (Indochina W2) of *P. falciparum* with IC_50_ values of 4.3, 6.0 and 7.7 µM, respectively. It was also found that carmabin A (**40**) was more cytotoxic to mammalian Vero cells (IC_50_ = 9.8 µM) than dragomabin (**41**) (IC_50_ = 182.3 µM) or dragonamide A (**42**) (IC_50_ = 67.8 µM), indicating that dragomabin (**41**) exhibited the best differential toxicity between parasitic and mammalian cells among the tested compounds in this series. The presence of three extra carbons in the aliphatic chain of carmabin A (**40**) may have contributed to its increased cytotoxicity over that displayed by dragomabin (**41**) [66].

**Figure 18:**
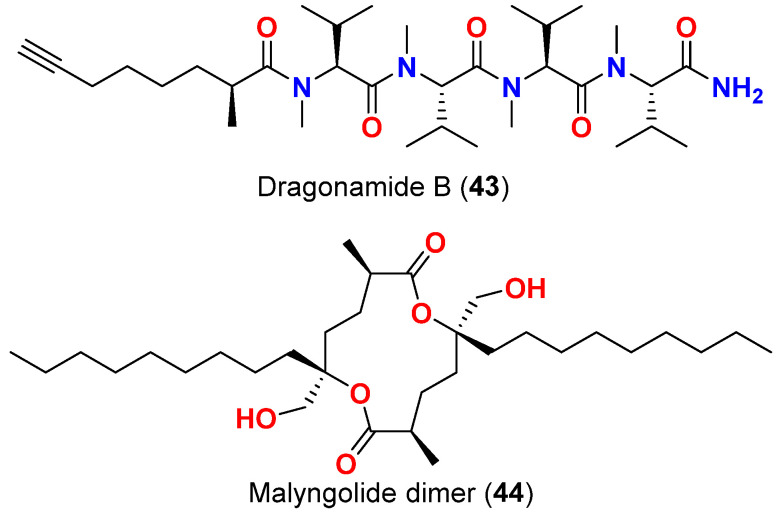
Chemical structures of compounds **33** and **34**.

On the other hand, the nonaromatic analog, dragonamide B (**43**) (Figure 18), was reported from a *L. majuscula* collected in Panama in 2007, was found to be completely inactive suggesting the necessity of an aromatic amino acid moiety at the carboxy terminus for the antimalarial activity [66]. Interestingly, when dragonamide A (**42**) was subjected to the same antimalarial assay on a later date, no activity was shown against the parasite (maximum test concentration 10 μM) [67].

In 2010, the antimalarial malyngolide dimer (**44**) (Figure 18), a symmetric cyclodepside isolated from *L. majuscula* in Coiba National Park, Panama was reported. It showed an IC_50_ value of 19 μM when tested against the chloroquine-resistant *P. falciparum* strain (W2) [68].

The intriguing cyclic depsipeptides lagunamides A-C (**21**–**23**) (Figure 9), purified from *L. majuscula* found in Pulau Hantu Besar, Singapore, also showed significant activity against the drug-sensitive NF54 strain of *P. falciparum*, with IC_50_ values of 0.19, 0.91 and 0.29 μM, respectively [50,51].

**Figure 19:**
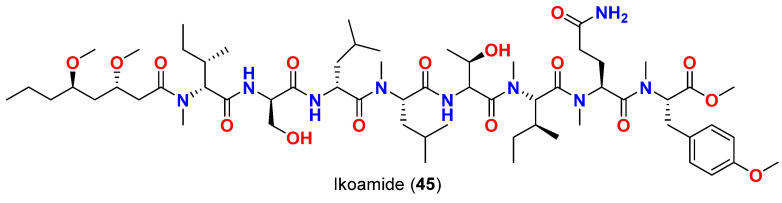
Chemical structure of compound **45**.

Ikoamide (**45**) (Figure 19), an antimalarial lipopeptide reported in 2020 from a marine cyanobacterium *Okeania* sp. collected in Okinawa, Japan. The compound displayed strong antimalarial activity against *P. falciparum* with an IC_50_ value of 0.14 μM without cytotoxicity against human cancer cell lines (HeLa and HL60) at 10 μM [69].

**Figure 20:**
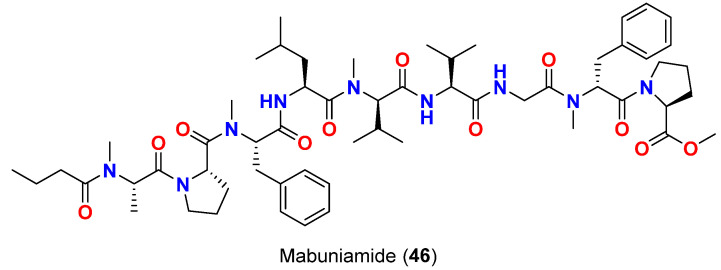
Chemical structure of compound **46**.

Mabuniamide (**46**) (Figure 20), a lipopeptide from of an Okinawan *Okeania* sp. in 2019 exhibited moderate antimalarial activity with IC_50_ of 1.4 μM against *P. falciparum* [70]. 

**Figure 21:**
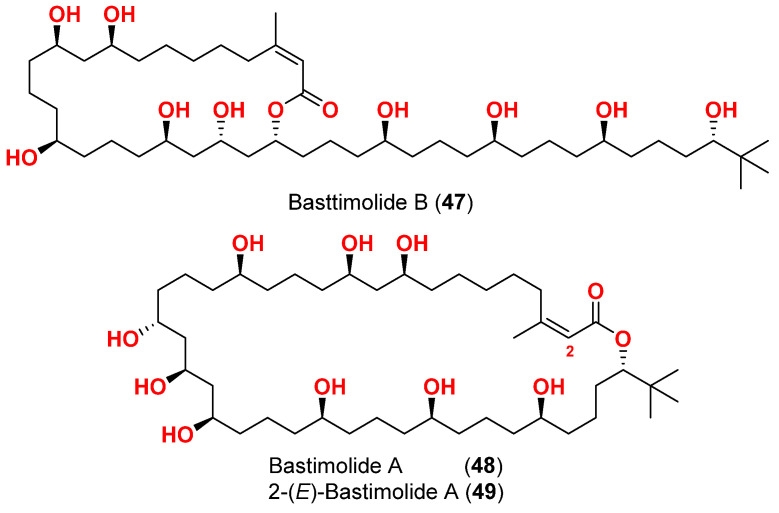
Chemical structures of compounds **47**–**49**.

Bastimolide B (**47**) (Figure 21), a 24-membered polyhydroxy macrolide with a long aliphatic polyhydroxylated side chain and unique terminal tertbutyl group was purified from *Okeania hirsuta* collected in Panama [71]. It showed a strong antimalarial activity against chloroquine-sensitive *P. falciparum* strain HB3 with IC_50_ of 5.7 µM.

On the other hand, bastimolide A (**48**) (Figure 21), which was obtained from the cyanobacterium *Okeania hirsuta* collected at the Caribbean coast of Panama, showed IC_50_ with 2.6 µM against chloroquine-sensitive *P. falciparum* strain [72]. Interestingly, 2-(*E*)-bastimolide A (**49**) (Figure 21), a methanolysis product of bastimolide A, displayed the greatest antimalarial activity with IC_50_ of 1.4 µM. It was found that, the existence of the double bond (at C-2/C-3) as well as the 1,3-diol (at C-9 and C-11) and 1,3,5-triol (at C-19, C-21, and C-23) functionalities were found to be important for the antimalarial activity [72].

**Figure 22:**
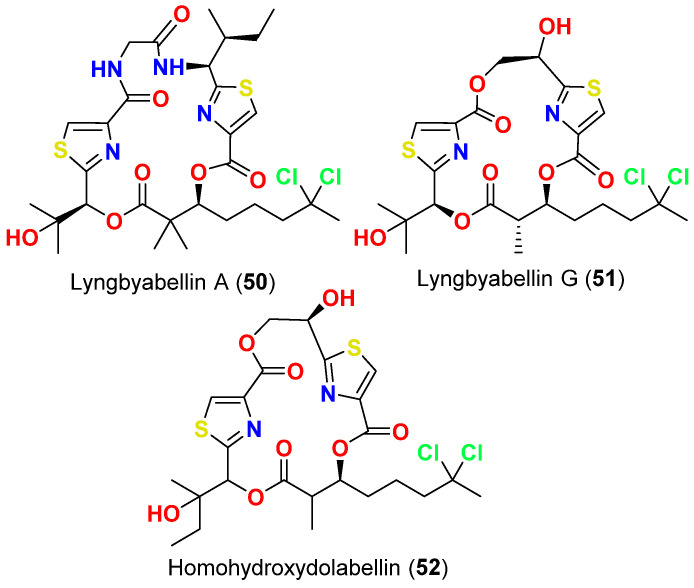
Chemical structures of compounds **50**–**52**.

In 2020, lyngbyabellins A (**50**) (Figure 22) was reported from the Malaysian *Moorea bouillonii*, while lyngbyabellein G (**51**) (Figure 22) was isolated from the Saudi Red Sea *Okeania* sp. Both compounds inhibited *P. falciparum* with IC_50_ of 0.3 and 1.1 µM, respectively [73].

On the other hand, homohydroxydolabellin (**52**) (Figure 22), which was isolated from the Malaysian *M. bouillonii* displayed IC_50_ of 6.4 µM against *P. falciparum* [73]. 

Another tropical parasite, which is the causative organism of the disease leishmaniasis, is *Leishmania donovani.* Antileishmanial activity has been displayed by a number of compounds isolated from *Lyngbya* sp.

**Figure 23:**
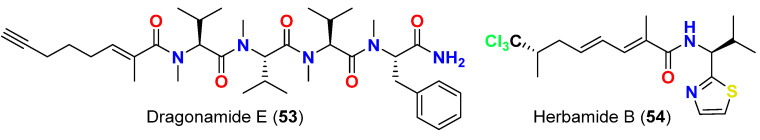
Chemical structures of compounds **53** and **54**.

Dragonamides A (**42**) (Figure 17) and E (**53**) (Figure 23) and herbamide B (**54**) (Figure 23), modified linear lipopeptides isolated in 2010 from Panamanian *L. majuscula* found around mangrove roots in the Bastimentos National Park, Bocas del Toro, Panama, showed inhibitory activities against *L. donovani* (LD-1S/MHOM/SD/00-strain 1S) with IC_50_ values of 6.5, 5.1 and 5.9 μM, respectively [67].

**Figure 24:**
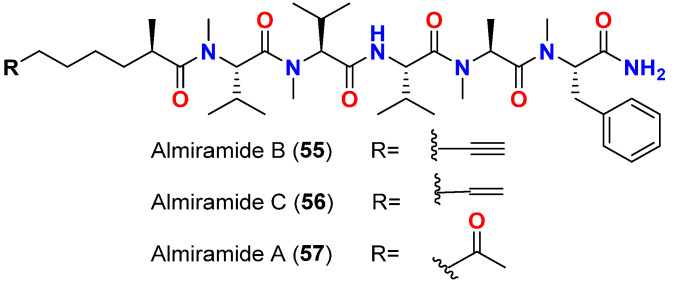
Chemical structures of compounds **55**–**57**.

Almiramides B (**55**) and C (**56**) (Figure 24), members of another class of linear lipopeptides isolated in 2010 from the Panamanian collection of the marine cyanobacterium *Lyngbya majuscula*, also exhibited antileishmanial activities, with IC_50_ values of 2.4 and 1.9 μM, respectively, whereas almiramide A (**57**) (Figure 24) was completely inactive up to 13.5 μM. This lack of activity might be attributed to the absence of an unsaturated terminus on the side chain, which was present in the active compounds, almiramides B (**55**) and C (**56**). Additionally, these compounds did not exert significant cytotoxicity to mammalian Vero cells and were selective for parasitic cells [74].

**Figure 25:**
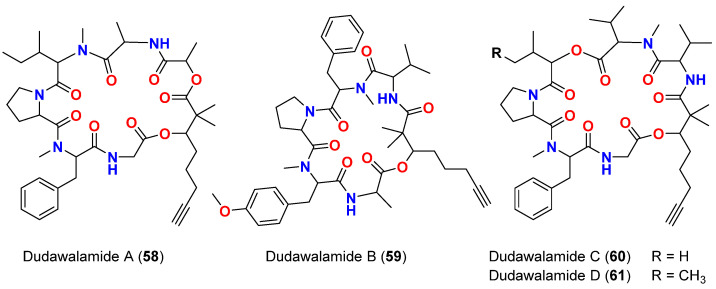
Chemical structures of compounds **58**–**61**.

The cyclic depsipeptides dudawalamides A-D (**58**–**61**) (Figure 25) are reported in 2017 from *M. producens* collected in Papua New Guinean *M* found in Dudawali Bay. The compounds exhibited broad and variable antiparasitic activities against malaria-, leishmaniasis- and Chagas disease-causing microorganisms (*P. falciparum, L. donovani* and *Trypanosoma cruzi*, respectively).

It was found that dudawalamides A (**58**) and D (**61**) were more potent against *P. falciparum* with IC_50_ values of 3.6 and 3.5 μM, respectively, compared to dudawalamides B (**59**) and C (**60**) (IC_50_ = 8.0 and 10 μM, respectively). Dudawalamides A (**58**) and B (**59**) possessed 12 and 7% growth inhibition at 10 μg/mL, respectively, against *T. cruzi*, and they both had an IC_50_ value > 10 μM against *L. donovani*. Dudawalamide D (**61**) was the most potent antiparasitic compound in this series since it exhibited an IC_50_ value of 2.6 μM against *L. donovani*, and inhibited *T. cruzi* by 60% when used at a concentration of 10 μg/mL [75].

It is interesting to note that cyclic depsipeptides with 2,2-dimethy-3-hydroxy-7-octynoic acid (Dhoya) moiety, which belong to the kulolide superfamily, possess only minor differences in structure and stereochemistry between each other; nevertheless, their potency was affected by such slight changes, indicating the significant role that configuration and residue sequence plays in the bioactivity of this class of compounds [75].

**Figure 26:**
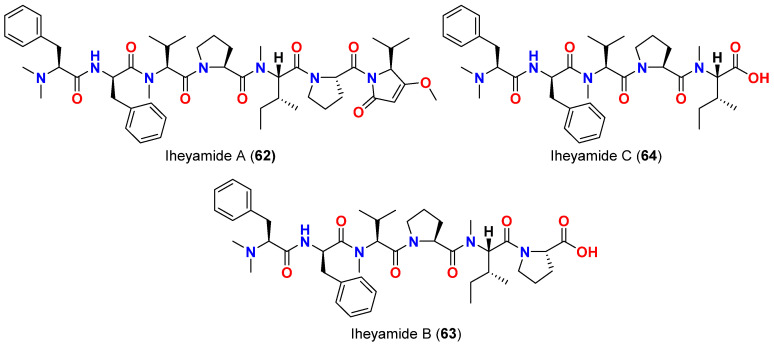
Chemical structures of compounds **62**–**64**.

In 2020, the linear peptides iheyamides A-C (**62**–**64**) (Figure 26) were reported from the cyanobacterium *Dapis* sp., collected in Okinawa, Japan [76]. Iheyamide A (**62**) showed moderate antitrypanosomal effect against *Trypanosoma brucei rhodesiense* and *T. bhurstuerusei brucei* with an IC_50_ value of 1.5 µM. It was found that the isopropyl-*O*-Me-pyrrolinone moiety is essential for the antitrypanosomal activity [76].

**Figure 27:**
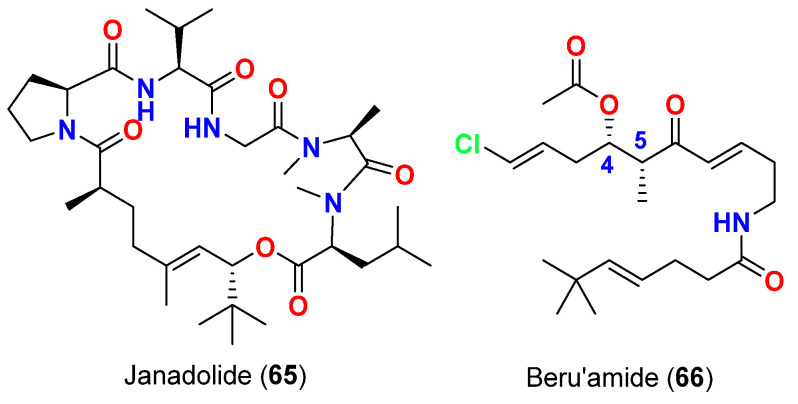
Chemical structures of compounds **65** and **66**.

In 2016, janadolide (**65**) (Figure 27), a cyclic polyketide−peptide hybrid with a *tert*-butyl group was reported from an *Okeania* sp., collected in Okinawa, Japan. The compound showed potent antitrypanosomal activity against *Trypanosoma brucei brucei* GUTat 3.1 strain with an IC_50_ value of 47 nM without cytotoxicity against human cells at 10 μM [77].

Finally, the polyketide beru’amide (**66**) (Figure 27) with 4*S*,5*R*-configuration was purified in very small amount (68 µg) from a cyanobacterium *Okeania* sp. collected in Kagoshima, Japan. Two synthetic enantiomers of beru’amide, 4*S*,5*R* and 4*R*,5*S*, were prepared and evaluated for their growth inhibition effects the causative parasite of African sleeping sickness *Trypanosoma brucei rhodesiensec* strains IL-1501. Interestingly, the enantiomers 4*S*,5*R* and 4*R*,5*S* of beru’amide displayed a closely similar and strong antitrypanosomal activity against *Trypanosoma brucei rhodesiense* with IC_50_ values of 1.2 and 1.0 μM, respectively. Accordingly, the absence of any noteworthy variance in the antitrypanosomal activities between the synthetic enantiomers, 4*S*,5*R* and 4*R*,5*S,* suggests that the absolute configurations are insignificant for the antitrypanosomal effect [78].

Table 5 displays all compounds with reported antiparasitic activities, their sources and collection sites as well as the targeted parasites and observed effects. 

## 7. Compounds with Antiviral Activities 

**Figure 28:**
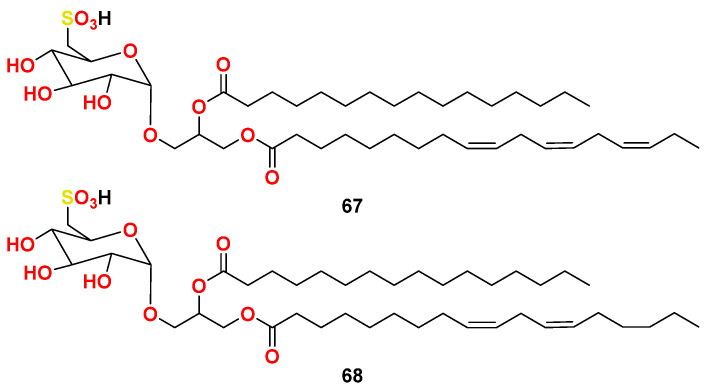
Chemical structures of compounds **67** and **68**.

Purification of the culture of the marine cyanobacterium *L. lagerheimii* that was collected in Hawaii resulted in the purification of two sulfoglycolipids (compounds **67** and **68**) (Figure 28). The compounds displayed activity against HIV-1 in cultured lymphoblastoid CEM, LDV-7, MT-2 and C3–44 cell lines in the tetrazolium assay and inp24 viral protein and syncytium formation assay [79]. The degree of inhibition HIV-1 by the compounds was generally comparable within a given cell line, but the degree of protection varied substantially among the different cell lines. The protective effects of the compounds were studied over a wide range of concentration range (about l–l00 µg/mL), depending on the target cell line and the mode of infection. Both compounds displayed similar levels of activity, suggesting that the length of the aliphatic side chain length and degree of unsaturation have no critical effect on the potency. Interestingly, sulfoglycolipids represent the first cyanobacterial derived compounds with antiviral activity [79].

In another studies, sulfoglycolipids inhibited the DNA polymerase function of the HIV-1 RT with IC_50_ values in the range 24–2950 nM without any significant effect on the ribonuclease H [80,81]. It was described that, the existence of a sulfate moiety in the sugar part as well as the aliphatic side chain are crucial for sulfoglycolipid’s effect on HIV RT [81].

Table 6 displays the compounds with reported antiviral activities, their sources and collection sites as well as the targeted viruses and observed effects. 

## 8. Compounds with Molluscicidal Anti-Diatoms Activities (Table 7)

Snails and slugs can damage crops by feeding on them; therefore, farmers and gardeners depend on molluscicides to protect their plants. There are some chemical compounds isolated from *Lyngbya* that possess molluscicidal activities.

Tanikolide (**34**), a lipid lactone was reported in 1999 from *L. majuscula* found in Tanikeli Island, Madagascar. The compound exhibited molluscicidal activity against the same snail (LD_50_ = 9.0 µg/mL) [62].

**Figure 29:**
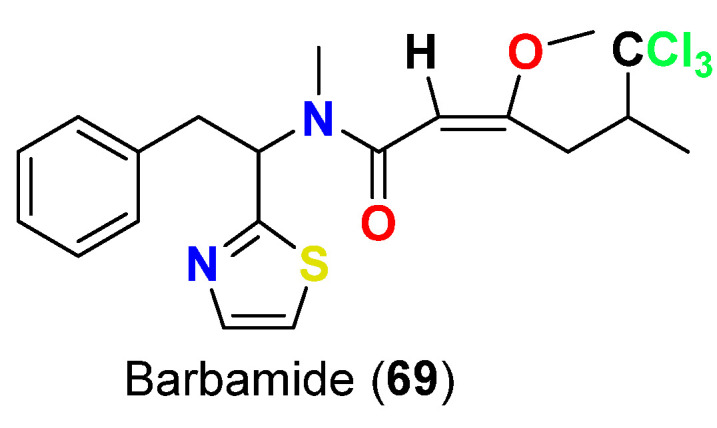
Chemical structure of compound **69**.

In addition, in 1996, a chlorinated lipopeptide, barbamide (**69**) (Figure 29), was reported from *L. majuscula* collected from Barbara Beach in Curaçao. It showed toxic effect on the mollusc *Biomphalaria glabrata* with LC_100_ of 10 µg/mL [82].

**Figure 30:**
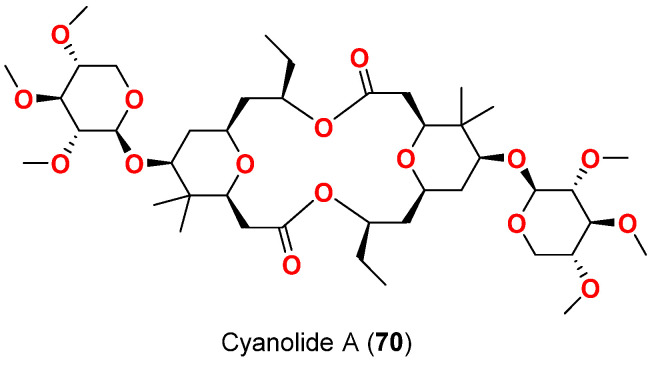
Chemical structure of compound **70**.

Finally, in 2010, the greatest potency of molluscicidal activity against *B. glabrata* was observed with cyanolide A (**70**) (Figure 30), a glycosidic macrolide isolated from Papua New Guinean *L. bouillonii* in Pigeon Island. The compound displayed molluscicidal effect with LC_50_ value against *B. glabrata* of 1.2 μM [83].

**Figure 31:**
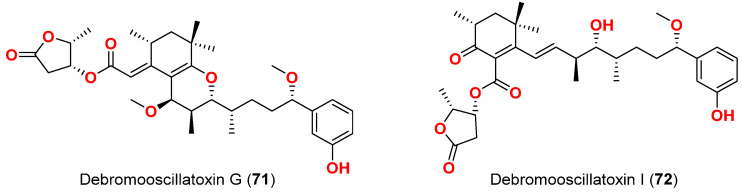
Chemical structures of compounds **71** and **72**.

In 2021, debromooscillatoxin G (**71**) and I (**72**) (Figure 31) were purified from an Okinawan cyanobacterium *Moorea prducens*. Both compounds moderately inhibited the growth of the marine diatom *Nitzschia amabilis* at a concentration of 10 µg/mL by 30% and 50%, respectively [84].

Table 7 displays the compounds with reported molluscicidal and anti-diatom activities, their sources and collection sites as well as the targeted organism and observed effects

**Table 7:** Compounds with reported molluscicidal and anti-diatom activities.

Compound	Source Organism	Collection Site	Targeted Organism	LC_50_/LC_100_/LD_50_/% of Inhibition	Reference
Tanikolide (**34**)	*L. majuscula*	Madagascar	*B. glabrata*	LD_50_ = 9.0 µg/mL	[62]
Barbamide (**69**)	*L. majuscula*	Curaçao	*B. glabrata*	LC_100_ = 10 µg/mL	[82]
Cyanolide A (**70**)	*L. bouillonii*	Papua New Guinea	*B. glabrata*	LC_50_ = 1.2 μM	[83]
Debromooscillatoxin G (**71**)	*M. producens*	Okinawa, Japan	*N. amabilis*	30% at 10 μg/mL	[84]
Debromooscillatoxin I (**72**)	*M. producens*	Okinawa, Japan	*N. amabilis*	30% at 10 μg/mL	[84]

## 9. Summary

**Figure 32:**
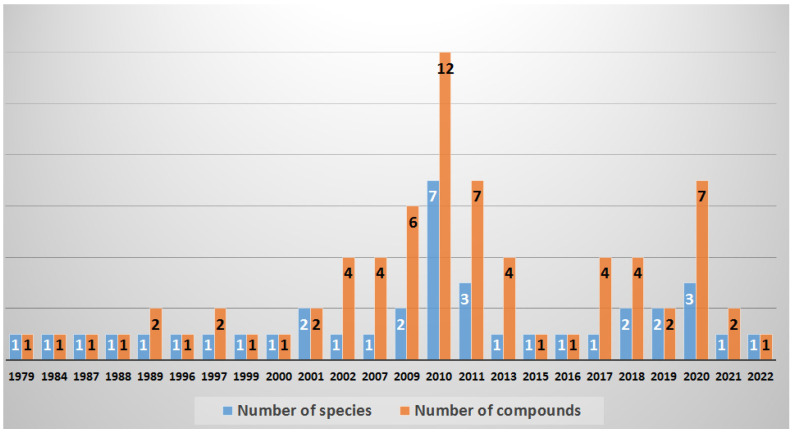
Number of investigated *Lyngbya*-morphotype and reported anti-infective compounds over time.

Secondary metabolites originating from the marine *Lyngbya* morphotype showed a huge chemical diversity and important biological activities, providing an unexploited potential for biodiscovery and therapeutics’ candidates. This marine-inspired genus *Lyngbya* has been a vital example since its first discovery back in 1979 as an untapped resource of marine-derived drug candidates. The existence of 72 compounds with anti-infective properties of marine derived *Lyngbya* morphotype worldwide (Figure 1), together with more than 40 years (Figure 32) of research efforts fashioned a resource empowering the biosynthetic capabilities of this genus. In aquatic environments, members of the marine derived *Lyngbya* morphotype have typically been obtained from different locations worldwide. Accordingly, the interest in marine derived *Lyngbya* species was growing, and became an essential source of chemical diversity with anti-infective effects.

Since the first report of the antibacterial malyngolide (**1**) in 1979, additional 71 compounds with anti-infective properties have been reported until now from 10 marine *Lyngbya* morphotype. The field was most active in the years 2002 (4 compounds from one species), 2007 (4 compound from one species), 2009 (6 compounds from 2 species), 2010 (12 compounds from 7 species), 2011 (7 compounds from 3 species), 2013 (4 compounds from one species), 2017 (4 compounds from one species), 2018 (4 compounds from 2 species), 2020 (7 compounds from 3 species), 2021 (2 compounds from one species) and finally in 2022 (one compound from one species) (Figure 32). Between 1979 and 2001 and in the years 2015, 2016, 2019, 2021 and 2022 there are reports about only one or two compounds from one or two species (Figure 32).

**Figure 33:**
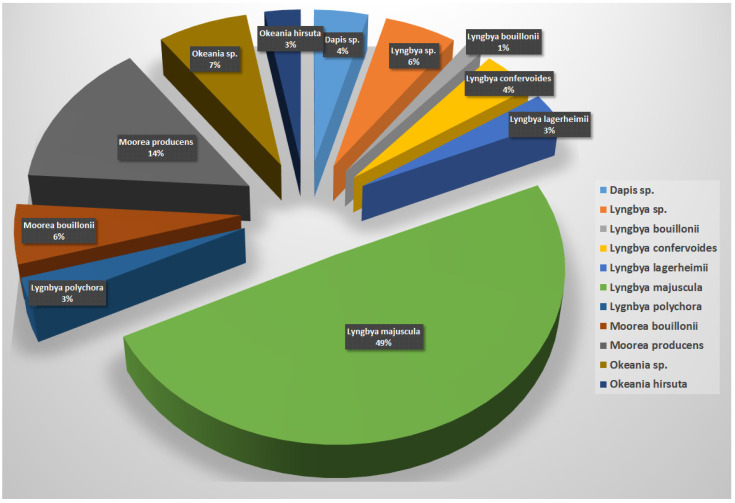
Number of reported anti-infective compounds per *Lyngbya* morphotype.

With regards to the source of the reported anti-infective compounds and as shown in Figure 33, it is clear that the morphotype *Lyngbya* is the main source of the compounds with 48 records (66%), followed by the morphotypes *Moorea* with 15 compounds (20%), *Okeania* with 9 compounds (10%) and *Dapis* with 3 compounds (4%) (Figure 33). Detailed contribution of the individual cyanobacterial morphotype is as follows: *Dapis* sp. (3 compounds), *Lyngbya* sp. (5 compounds), *Lyngbya confervoides* (3 compounds), *Lyngbya lagerheimii* (one compound), *Lyngbya majuscula* (37 compounds), *Lygnbya polychora* (2 compounds), *Moorea bouilloni* (4 compounds), *Moorea producens* (8 compounds), *Okeania* sp. (4 compounds and finally *Okeania hirsuta* (3 compounds) (Figure 33).

**Figure 34:**
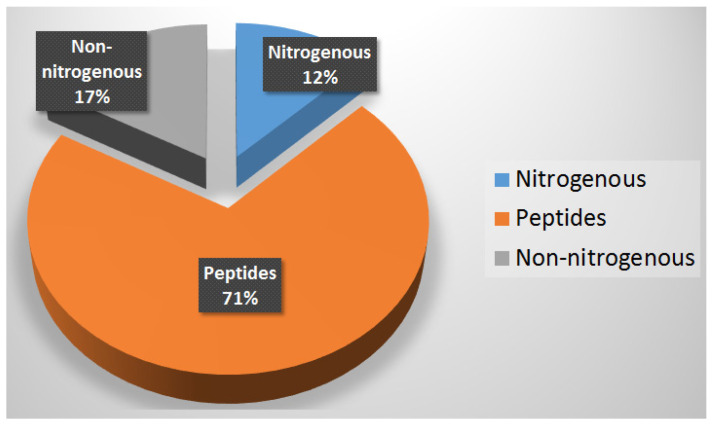
Distribution of the nitrogenous and non-nitrogenous compounds in *Lyngbya* morpho-type.

As per the chemical diversity of the genus *Lyngbya*, it could be noticed that nitrogenous compounds represented as a predominant class of reported secondary metabolites with 59 nitrogenous compounds (83%) and 12 non-nitrogenous compounds (17%). This existence of these enormous nitrogenous secondary metabolites could be attributed to the capability of the members of cyanobacteria of fixing atmospheric nitrogen. Peptides are represented by 71% (51 compounds) from the nitrogen-containing secondary metabolites, while regular nitrogenous compounds, including alkaloids and others are represented by 9 compounds (12%) (Figure 34). Interestingly, there are 14 halogenated compounds among the reported anti-infective secondary metabolites.

**Figure 35:**
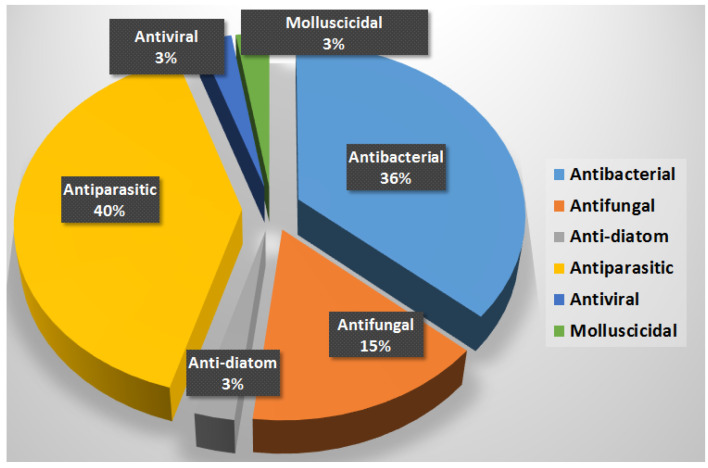
Number of reported compounds associated with biological activities.

Most *Lyngbya*-derived compounds have demonstrated excellent antibacterial and antiprotozoal activities against different pathogens and parasites. Out of the 72 reported secondary metabolites from *Lygnbya* morphotype, 31 compounds (about 40%) have been reported to possess antiparasitic activities. In addition, 28 compounds (36%) of the reported compounds displayed antibacterial effects. With antifungal effects, the number was much less with only 12 compounds (15%). Finally, 3 compounds contributed to molluscicidal activity, 2 compounds for each of the antiviral and anti-diatom effects (Figure 35).

## 10. Conclusions

Herein, 72 compounds, mostly peptides, derived from different *Lyngbya* morphotype are described. To the best of our knowledge, the anti-infective compounds in this review showed significant activities, including antibacterial, anti-swarming, ant-quorum sensing, antifungal, antiparasitic, antiviral and molluscicidal activities. Therefore, members of the genus *Lyngbya* morphotype represent a therapeutic gold mine of chemically and biologically diverse natural products that exhibit a wide array of anti-infective effects. The isolation of these chemical compounds over the span of more than forty years and the compounding evidence collected from biological and pharmacological investigations in support of the compounds’ pharmaceutical potential makes this intriguing cyanobacterium a significant target for biomedical research and novel drug leads development. Therefore, special attention should be given to the original source of such compounds when searching for medically or environmentally useful natural products. Therefore, a potential way to drug development from the marine cyanobacterium *Lyngbya* would be the optimization of its cultivation in the laboratory under the condition which would optimize the production of the desired biologically active metabolites. Due to the special supplies, which are required not only for cyanobacterial growth but also for the optimization of the production of cyanobacterial secondary metabolites, broad efforts are worried with this approach.

In summary, members of the *Lyngbya* morphotype have been exceptional sources of biosynthetic and biochemical novelty applied to drug discovery. Even facing significant headwinds, new discoveries from *Lyngbya* morphotype continue apace.

## Figures and Tables

**Table 1:** Number of reported anti-infective compounds from *Lyngbya* morphotype.

Species Name ^a^	Number of Reported Compounds
*Dapis* sp.	3
*Lyngbya* sp.	4
*Lyngbya bouillonii*	1
*Lyngbya confervoides*	3
*Lyngbya lagerheimii*	2
*Lyngbya majuscula*	35
*Lygnbya polychora*	2
*Moorea bouillonii*	4
*Moorea producens*	11
*Okeania* sp.	5
*Okeania hirsuta*	2

^a^ Names are given as reported in the original manuscripts.

**Table 2:** Compounds with reported antibacterial activities.

Compound	Source Organism	Collection Site	Targeted Bacteria	MIC/Inhibition Zone/IC_50_	Reference
Malyngolide (**1**)	*L. majuscula*	Hawaii, USA	*M. smegmatis, S. pyogenes, S. aureus* and *B. subtilis*	More active against *M. smegmatis* and *S. pyogenes* than *S. aureus* and *B. subtilis*	[40]
Lyngbic acid (**2**)	*M. producens*	Red Sea	*M. tuberculosis* H_37_Rv	65% inhibition at 12.5 μg/mL	[41]
Lyngbic acid (**2**)	*L. majuscula*	Caribbean region	*S. aureus* and *B. subtilis*	Antibacterial activity	[42]
Malyngamide D acetate (**3**)	*L. majuscula*	Caribbean region	*S. aureus*	Slight activity	[42]
Pitipeptolide A (**4**)	*L. majuscula*	Guam	*M. tuberculosis* ATCC 25177	25 mm at 100 µg 10 mm at 25 µg	[43]
Pitipeptolide A (**4**)	*L. majuscula*	Guam	*M. tuberculosis* ATCC 35818	15 mm at 100 µg 9 mm at 25 µg	[43]
Pitipeptolide B (**5**)	*L. majuscula*	Guam	*M. tuberculosis* ATCC 25177	30 mm at 100 µg 15 mm at 25 µg	[43]
Pitipeptolide B (**5**)	*L. majuscula*	Guam	*M. tuberculosis* ATCC 35818	15 mm at 100 µg 10 mm at 25 µg	[43]
Pitipeptolide A (**4**)	*L. majuscula*	Guam	*M*. *tuberculosis* ATCC 25177	28 mm at 100 µg 23 mm at 50 µg 9 mm at 10 µg	[44]
Pitipeptolide B (**5**)	*L. majuscula*	Guam	*M*. *tuberculosis* ATCC 25177	30 mm at 100 µg 24 mm at 50 µg 14 mm at 10 µg	[44]
Pitipeptolide C (**6**)	*L. majuscula*	Guam	*M*. *tuberculosis* ATCC 25177	26 mm at 100 µg 21 mm at 50 µg 18 mm at 10 µg	[44]
Pitipeptolide D (**7**)	*L. majuscula*	Guam	*M*. *tuberculosis* ATCC 25177	10 mm at 100 µg 0 mm at 50 µg 0 mm at 10 µg	[44]
Pitipeptolide E (**8**)	*L. majuscula*	Guam	*M*. *tuberculosis* ATCC 25177	21 mm at 100 µg 15 mm at 50 µg 0 mm at 10 µg	[44]
Pitipeptolide F (**9**)	*L. majuscula*	Guam	*M*. *tuberculosis* ATCC 25177	40 mm at 100 µg 30 mm at 50 µg 10 mm at 10 µg	[44]
Pitiprolamide (**10**)	*L. majuscula*	Guam	*M. tuberculosis* ATCC 25177	23 mm at 100 µg 13 mm at 50 µg 0 mm at 10 µg	[45]
Pitiprolamide (**10**)	*L. majuscula*	Guam	*B. cereus* ATCC 10987	IC_50_ = 70 μM at 1 μM	[45]
Mixture of lyngbyazothrins A and B (**14** and **15**)	*Lyngbya* sp.	Germany (Culture)	*M. flaVus* SBUG 16	8 mm at 100 μg/disk	[46]
Mixture of lyngbyazothrins C (**16**) and D (**17**)	*Lyngbya* sp.	Germany (Culture)	*B. subtilis* SBUG 14 *E. coli* ATCC 11229 *E. coli* SBUG 13 *P. aeruginosa* ATCC 27853 *S. marcescens* SBUG 9	18 mm at 25 μg/disk 18 mm at 100 μg/disk 15 mm at 100 μg/disk 8 mm at 100 μg/disk 8 mm at 200 μg/disk	[46]
Tiahuramide A (**18**)	*L. majuscula*	French Polynesia	*A. salmonicida* (CIP 103209T strain), *V. anguillarum* (CIP 63.36T), *S. baltica* (CIP 105850T), *E. coli* (CIP 54.8) and *M. luteus* (CIP A270)	MIC = 27, 33, >50, 35 and 47 μM, respectively	[47]
Tiahuramide B (**19**)	*L. majuscula*	French Polynesia	*A. salmonicida* (CIP 103209T strain), *V. anguillarum* (CIP 63.36T), *S. baltica* (CIP 105850T), *E. coli* (CIP 54.8) and *M. luteus* (CIP A270)	MIC = 9.4, 8.5, 22, 12 and 29 μM, respectively	[47]
Tiahuramide C (**20**)	*L. majuscula*	French Polynesia	*A. salmonicida* (CIP 103209T strain), *V. anguillarum* (CIP 63.36T), *S. baltica* (CIP 105850T), *E. coli* (CIP 54.8) and *M. luteus* (CIP A270)	MIC = 6.7, 7.4, 16, 14 and 17 μM, respectively	[47]

**Table 3:** Compounds with reported anti-swarming and anti-quorum sensing activities.

Compound	Source Organism	Collection Site	Targeted Bacteria/Receptor	Anti-Swarming/Anti-Quorum Sensing	Reference
Lagunamide A (**21**)	*L. majuscula*	Singapore	*P. aeruginosa* PA01	Anti-swarming effect: 62% at 100 ppm	[50,51]
Lagunamide B (**22**)	*L. majuscula*	Singapore	*P. aeruginosa* PA01	Anti-swarming effect: 56% at 100 ppm	[50,51]
Lagunamide C (**23**)	*L. majuscula*	Singapore	*P. aeruginosa* PA01	Anti-swarming effect: 49%, at 100 ppm	[50,51]
Malyngamide C (**24**)	*L. majuscula*	Florida, USA	3-oxo-C_12_-HSL (*N*-3-oxo-dodecanoyl-L-homoserine lactone) signaling in a LasR-based quorum sensing (QS) reporter pSB1075	QS inhibitor reduction in 3-oxo-C_12_-HSL signaling at 10, 100 and 1000 µM	[54]
8-*epi*-Malyngamide C (**25**)	*L. majuscula*	Florida, USA	3-oxo-C_12_-HSL (*N*-3-oxo-dodecanoyl-L-homoserine lactone) signaling in a LasR-based quorum sensing (QS) reporter pSB1075	QS inhibitor reduction in 3-oxo-C_12_-HSL signaling at 10, 100 and 1000 µM	[54]
Malyngolide (**1**)	*L. majuscula*	Florida, USA	Production of violacein pigment by *C. violaceum* CV017 in the QS bioassay	QS inhibitor inhibition of violacein production with effective concentrations ranged from 0.07 to 0.22 mM; EC_50_ = 0.11 mM	[55]
			Responses of *lasR*^+^P_lasI_-*luxCDABE* reporter pSB1075 in the presence of 14 µM of 3-oxo-C_12_-HSL	Inhibition of responses of the *lasR*^+^P_lasI_-*luxCDABE* reporter pSB1075 with concentrations ranging from 3.57 to 57; EC_50_ = 12.2 µM	[55]
			Production of elastase by *P. aeruginosa* PAO1 (an extracellular enzyme regulated by 3-oxo-C_12_-HSL and LasR)	Significant reduction in elastase production; EC_50_ = 10.6 µM, at higher concentrations of MAL, elastase production was inhibited to the level observed in the QS mutant of *P. aeruginosa* JP2	[55]
Lyngbyoic acid (**26**)	*L. majuscula*	Florida, USA	Four reporters based on different acylhomoserine lactone (AHL) receptors acylhomoserine lactone (AHL) receptors (LuxR, AhyR, TraR and LasR)	QS inhibitor, most effective inhibition against LasR reporter	[56]
			Production of pyocyanin and elastase (LasB) both on the protein and transcript level in wild-type *P. aeruginosa.*	Reduction in the production of pyocyanin and elastase (LasB) and direct inhibition of LasB enzymatic activity; *K*_i_ = 5.4 mM	
Doscadenamide A (**27**)	*L. bouillonii*	Guam	3-Oxo-C_12_-HSL-responsive reporter plasmid pSB1075, which encodes LasR and contains a light-producing *luxCDABE* cassette expressed in *E. coli*	QS agonist in a LasR-dependent manner and activation of 3-oxo-C_12_-HSL-responsive reporter plasmid pSB1075	[57]
			Production of QS pigment pyocyanin in wild-type *P. aeruginosa*	Increase pyocyanin production at 10 µM	

**Table 4:** Compounds with reported antifungal activity.

Compound	Source Organism	Collection Site	Targeted Fungi	MIC/Inhibition Zone/LD_50_	Reference
Majusculamide C (**28**)	*L. majuscula*	Marshall Islands	*P. infestans* and *P. viticola*	Growth inhibition	[58]
57-Normajusculamide C (**29**)	*L. majuscula*	Marshall Islands	*S. pastorianus*	Antimycotic activity	[59]
Microcolin A (**30**)	*L. polychroa*	Marshall Islands	*D. salina* (SIO and EBGJ strains)	LD_50_ = >200 μg/mL	[60]
Microcolin B (**31**)	*L. polychroa*	Marshall Islands	*D. salina* (SIO and EBGJ strains)	LD_50_ = >200 μg/mL	[60]
Laxaphycin B (**32**)	*L. majuscula*	French Polynesia	*C. albicans*	Antifungal activity	[61]
Mixture of laxaphycins A (**33**) and B (**32**)	*L. majuscula*	French Polynesia	*C. albicans*	Laxaphycin B produces synergetic effect to the inactive laxaphycin A	
Tanikolide (**34**)	*L. majuscula*	Madagascar	*C. albicans*	13 mm at 100 µg/disk	[62]
Lyngbyabellin B (**35**)	*L. majuscula*	Florida, USA	*C. albicans* (ATCC 14053)	10.5 mm at 100 µg/disk	[63]
Hectochlorin (**36**)	*L. majuscula*	Jamaica	*C. albicans* (ATCC 14053)	16 mm at 100 µg/disk 11 mm at 10 µg/disk	[64]
Lobocyclamide A (**37**)	*L. confervoides*	Southern Bahamas	*C. albicans* 96–489 (Fluconazole-resistant)	7 mm at 150 µg/disk and MIC = 100 µg/mL	[65]
Lobocyclamide B (**38**)	*L. confervoides*	Southern Bahamas	*C. albicans* 96–489 (Fluconazole-resistant)	8 mm at 150 µg/disk and MIC = 30–100 µg/mL	[65]
Lobocyclamide B (**38**)	*L. confervoides*	Southern Bahamas	*C. glabrata*	6 mm at 150 µg/disk	[65]
Mixture of lobocyclamides A and B (**37** and **38**)	*L. confervoides*	Southern Bahamas	-	MIC = 10–30 µg/mL	[65]
Lobocyclamide C (**39**)	*L. confervoides*	Southern Bahamas	*C. albicans* 96–489 (Fluconazole-resistant)	10 mm at 150 µg/disk	[65]
Lobocyclamides C (**39**)	*L. confervoides*	Southern Bahamas	*C. glabrata*	8 mm at 150 µg/disk	[65]

**Table 5:** Compounds with reported antiparasitic activities.

Compound	Source Organism	Collection Site	Targeted Microbe/Parasite	IC_50_/% of Inhibition	Reference
Lagunamide A (**21**)	*L. majuscula*	Singapore	*P. falciparum* (NF54 strain)	IC_50_ = 0.19 μM	[50,51]
Lagunamide B (**22**)	*L. majuscula*	Singapore	*P. falciparum* (NF54 strain)	IC_50_ = 0.91 μM	[50,51]
Lagunamide C (**23**)	*L. majuscula*	Singapore	*P. falciparum* (NF54 strain)	IC_50_ = 0.29 μM	[50,51]
Carmabin A (**40**)	*L. majuscula*	Panama	*P. falciparum* (Indochina W2 strain)	IC_50_ = 4.3 µM	[66,67]
Dragomabin (**41**)	*L. majuscula*	Panama	*P. falciparum* (Indochina W2 strain)	IC_50_ = 6.0 µM	[66,67]
Dragonamide A (**42**)	*L. majuscula*	Panama	*P. falciparum* (Indochina W2 strain)	IC_50_ = 7.7 µM	[66,67]
Dragonamide A (**42**)	*L. majuscula*	Panama	*L. donovani* (LD-1S/MHOM/SD/00-strain 1S)	IC_50_ = 6.5 μM	[67]
Malyngolide dimer (**44**)	*L. majuscula*	Panama	*P. falciparum* (W2 strain)	IC_50_ = 19 μM	[68]
Dragonamide E (**53**)	*L. majuscula*	Panama	*L. donovani* (LD-1S/MHOM/SD/00-strain 1S)	IC_50_ = 5.1 μM	[67]
Herbamide B (**54**)	*L. majuscula*	Panama	*L. donovani* (LD-1S/MHOM/SD/00-strain 1S)	IC_50_ = 5.9 μM	[67]
Almiramide B (**55**)	*L. majuscula*	Panama	*L. donovani* (LD-1S/MHOM/SD/00-strain 1S)	IC_50_ = 2.4 μM	[74]
Almiramide C (**56**)	*L. majuscula*	Panama	*L. donovani* (LD-1S/MHOM/SD/00-strain 1S)	IC_50_ = 1.9 μM	[74]
Dudawalamide A (**58**)	*M. producens*	Papua New Guinea	*P. falciparum*	IC_50_ = 3.6 μM	[75]
Dudawalamide A (**58**)	*M. producens*	Papua New Guinea	*T. cruzi*	12% inhibition at 10 μg/mL	[75]
Dudawalamide A (**58**)	*M. producens*	Papua New Guinea	*L. donovani*	IC_50_ = >10 μM	[75]
Dudawalamide B (**59**)	*M. producens*	Papua New Guinea	*P. falciparum*	IC_50_ = 10 μM	[75]
Dudawalamide B (**59**)	*M. producens*	Papua New Guinea	*T. cruzi*	7% inhibition at 10 μg/mL	[75]
Dudawalamide B (**59**)	*M. producens*	Papua New Guinea	*L. donovani*	IC_50_ >10 μM	[75]
Dudawalamide C (**60**)	*M. producens*	Papua New Guinea	*P. falciparum*	IC_50_ = 3.5 μM	[75]
Dudawalamide D (**61**)	*M. producens*	Papua New Guinea	*P. falciparum*	IC_50_ = 8.0 μM	[75]
Dudawalamide D (**61**)	*M. producens*	Papua New Guinea	*T. cruzi*	60% inhibition at 10 μg/mL	[75]
Dudawalamide D (**61**)	*M. producens*	Papua New Guinea	*L. donovani*	IC_50_ = 2.6 μM	[75]
Iheyamide A (**62**)	*Dapis* sp.	Okinawa, Japan	*T. brucei rhodesiense* *T. bhurstuerusei brucei*	IC_50_ = 1.5 μM IC_50_ = 1.5 μM	[76]
Janadolide (**65**)	*Okeania* sp.	Okinawa, Japan	*T. brucei brucei*	IC_50_ = 47 nM	[77]
Beru’amide (**66**)	*Okeania* sp.	Kagoshima, Japan	*T. brucei rhodesiense*	IC_5_ = 1.2 μM	[78]

**Table 6:** Compounds with reported antiviral activities.

Compound	Source Organism	Collection Site	Targeted Virus	IC_50_/% of Inhibition	Reference
**67**	*L. lagerheimii*	Hawaii, USA	HIV-1	HIV-1 inhibition at l-l00 µg/mL	[79]
**68**	*L. lagerheimii*	Hawaii, USA	HIV-1	HIV-1 inhibition at l-l00 µg/mL	[79]

## Data Availability

Not applicable.
